# Novel approaches to enable equitable access to monoclonal antibodies in low- and middle-income countries

**DOI:** 10.1371/journal.pgph.0003418

**Published:** 2024-07-01

**Authors:** Shelly Malhotra, Anne-Isabelle Cameron, Dzintars Gotham, Esteban Burrone, Peter J. Gardner, Colleen Loynachan, Sébastien Morin, Cherise P. Scott, Carmen Pérez-Casas

**Affiliations:** 1 Global Access, IAVI, New York, New York, United States of America; 2 Strategy, Unitaid, Geneva, Switzerland; 3 Consultant, Gotham Research Ltd, London, United Kingdom; 4 Strategy, Policy and Market Access, Medicines Patent Pool, Geneva, Switzerland; 5 Infectious Disease Programme, Wellcome, London, United Kingdom; McGill University, CANADA

## Abstract

Monoclonal antibodies (mAbs) are revolutionizing management of non-communicable diseases in high-income countries and are increasingly being advanced for a range of infectious diseases (IDs). However, access to existing mAbs is limited in low- and middle-income countries (LMICs), and investment in developing fit-for-purpose mAbs for IDs that disproportionately affect LMICs has been limited. Underlying these access barriers are systemic challenges, including a lack of commercial incentives to target LMIC markets and complexity in manufacturing and regulatory processes. Novel strategies are needed to overcome systemic access barriers for mAbs. We outline key areas where new approaches could address these barriers, based on a multistakeholder consultation in March 2023. Three disease-market archetypes are identified to guide thinking about business models tailored to different contexts. New business models are needed to incentivize development and manufacturing of ID mAbs and to ensure mAbs are optimized with a target product profile and cost of goods that enable use in diverse LMIC settings. Lessons can be applied from voluntary licensing strategies and product development partnerships that have shown success in catalysing development and affordable supply for a range of infectious diseases. Technology transfer will be key to expand LMIC research and manufacturing capacity and to enable sustainable and diversified supply. Improved market intelligence, demand aggregation mechanisms, and portfolio-based manufacturing models could be used to de-risk commercial investment and establish a sustainable manufacturing ecosystem for affordable mAbs. Novel regulatory approaches and robust technology transfer may reduce data requirements and timelines for biosimilar approvals. Trailblazer products, with coordinated “end-to-end” support from funders, can demonstrate proof of concept for pathways to accessible mAbs across a broader range of LMICs. Research funders; local, regional, global health agencies; and, private sector partners should commit to implementing innovative partnerships and end-to-end strategies that enable equitable access to mAbs for infectious diseases in LMICs.

## Introduction

Monoclonal antibodies (mAbs) represent one of the most important medical innovations in modern pharmacology. mAbs are lab-derived versions of the antibodies produced by the immune system. mAbs can be designed to act on a wide range of pharmacological targets, which has enabled the development of mAbs to tackle diseases that were previously difficult or impossible to treat [1]. mAbs make up a steadily increasing proportion of new medicines, comprising 27% of new U.S. Food and Drug Administration (FDA) drug approvals in 2022 [2, 3].

mAbs have become the standard of care for many cancers and autoimmune diseases in high-income countries (HICs). Additionally, they show considerable promise for the treatment and prevention of many infectious diseases (IDs), having a unique and complementary role with vaccines and small-molecule medicines across a range of use-cases. Multiple international organisations working in global health have highlighted the potential of mAbs to address a range of infectious diseases, including for pandemic preparedness [1, 4–9].

However, the current mAbs development pipeline for infectious diseases remains insufficient. Of the first 100 antibody-based drugs approved in the US, only 7 were for an ID indication [3], and access to mAbs is highly unequal across countries. Roughly 80% of sales for mAbs are in HICs.^1^ Inversely, Africa represents only roughly 1% of global sales, despite being home to a disproportionate burden of disease from a range of applicable noncommunicable diseases (NCDs) and IDs [1]. Some LMICs, such as India, Brazil, and Egypt, have expanded their domestic mAbs manufacturing capacity [10]. Where mAbs are available in LMICs, however, they are often not integrated into national insurance schemes or essential medicine lists, limiting access [1]. High prices present a key barrier to accessing mAbs, with many priced at tens or hundreds of thousands of US dollars per year [1, 11].

Palivizumab, used in the prevention of respiratory syncytial virus (RSV), was the first mAb to be approved for an ID indication in 1998. However, access to palivizumab, as well as to a next-generation mAb for RSV (nirsevimab), remains low in LMICs due to high prices and limited global supply [1, 12–14]. While mAbs are increasingly being included in the World Health Organization (WHO) Model List of Essential Medicines, mainly for cancer indications, several have been rejected due to their prohibitively high prices [15].

## Key challenges and enablers for advancing access to mAbs for infectious diseases

With promising mAbs now available or in the pipeline for a range of IDs that disproportionately affect LMICs, overcoming barriers to access will be critical. Underlying gaps in research, development and access to forthcoming mAbs are numerous systemic challenges, including ill-adapted target product profiles, weak economic incentives, business models that are misaligned with global health needs, high development and manufacturing costs, regulatory hurdles, and gaps in end-to-end funding for mAbs. These issues must urgently be addressed to catalyse timely and equitable access to already approved mAbs, as well as those in the pipeline.

In this article, we explore potential barriers to increasing access to ID mAbs and outline key recommendations to address these challenges across four domains: 1) Increasing research and development (R&D) and innovations in manufacturing for optimized mAbs; 2) Intellectual property (IP) management, voluntary licensing, and technology transfer; 3) Regulatory pathways and strategies to facilitate mAbs availability; and 4) Market shaping, de-risking, and demand creation.

### Increasing R&D and manufacturing innovation for optimized mAbs

#### Challenge

**Fig 1:**
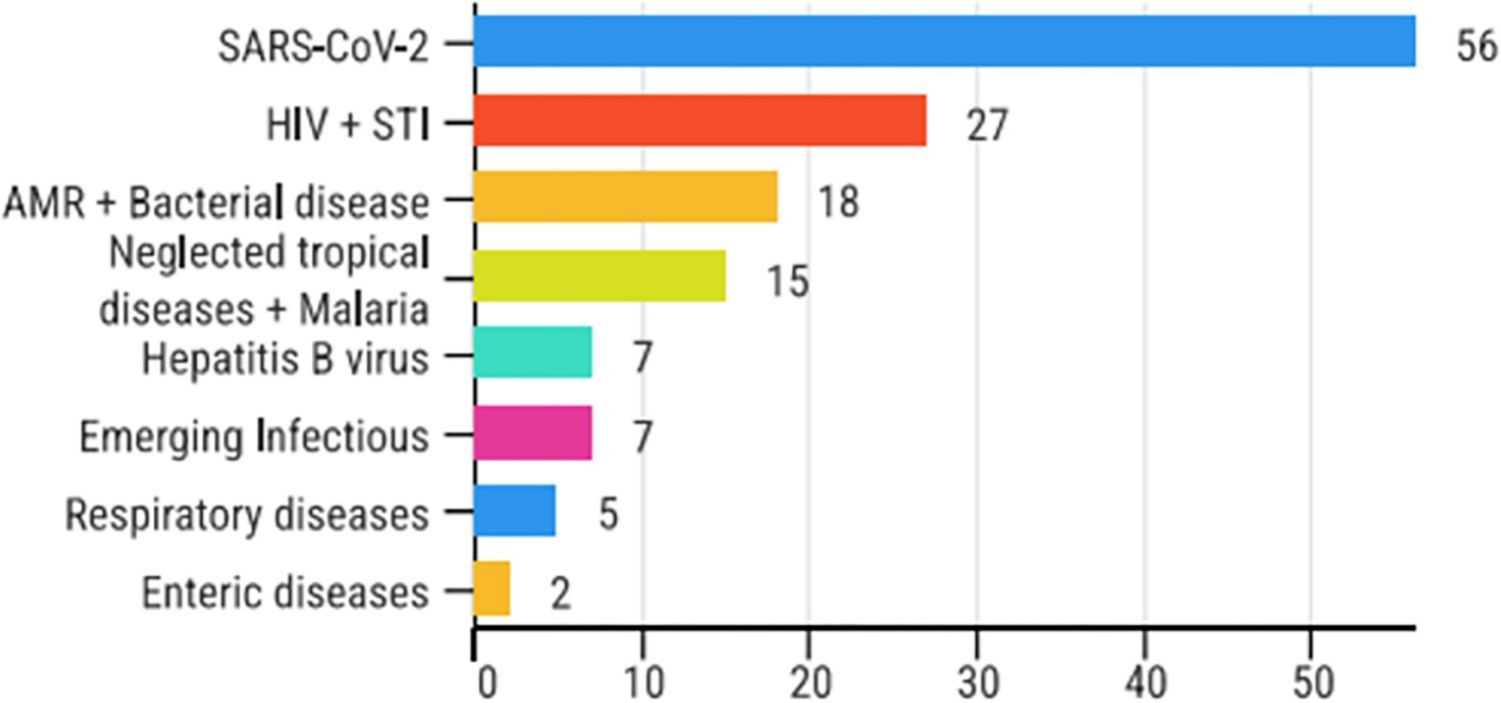
Infectious disease mAbs in the clinical development pipeline. Source: Gieber, L. IAVI pipeline review based on Clinicaltrials.gov, Pan Africa Clinical Trials Registry, the Antibody Society, and various manufacturers’ websites.

To date, 18 mAbs have been approved for diverse ID indications, and over 130 ID mAb products are in clinical development (*Fig 1*). However, this pipeline is modest compared to other disease areas [16], reflecting the broader withdrawal of large pharmaceutical companies from ID R&D over the past three decades [17, 18], and limited attention to neglected diseases, which make up only 0.3% of the pharmaceutical industry’s total R&D investments [19].

While R&D investment in ID mAbs has increased each year since 2016 [20], it has largely been focused on developing biodefense and outbreak pathogen products for stockpiling and treatment use-cases for HICs, which has not yielded broad access to innovations [21]. There has been limited investment in mAb product development and optimization for use in LMICs.

Manufacturing of mAbs is significantly more complex than manufacturing small molecules, involving a larger number of steps and specialized input materials, workforces, and facilities. These factors contribute to a higher costs of goods sold (COGS).

COGS are decreasing with the optimization of product profiles for increased potency and half-life, innovations in mAbs manufacturing, and large-scale production. While average COGS were around $300/g In the early 2000s [22, 23], they are currently estimated to be around US$100/g or less. For well-established, highly productive antibodies produced at very large scale (VLS), COGS of <$50/g for bulk drug substance are currently achievable [22, 24, 25].

#### Key enablers

*Coordination of funding*. Coordination amongst funders to support different parts of the ‘end-to-end’ continuum from R&D to last-mile delivery will be important. Funders should apply leverage to ensure accessibility considerations are factored early-on in the R&D process.

*Involving LMIC governments*, *researchers*, *and communities early in development*. The design and governance of initiatives to expand R&D for, and access to, ID mAbs should involve relevant communities and LMIC governments as active partners to ensure mAbs meet the needs and preferences of end-users and health systems. To strengthen the role of communities and end-users, priority-setting initiatives should harness diverse perspectives through multistakeholder consultation. WHO has used such approaches to develop preferred product characteristics (PPCs) guidance for mAbs for the prevention of malaria, HIV, and RSV [4–6, 26]. Consultative mechanisms should be complemented by robust research on user preferences and feasibility considerations for mAbs delivery across a range of LMIC settings [27].

*Product and manufacturing optimization to lower COGS*. Novel technologies and platforms are being explored to lower COGS. Most mAbs are manufactured in Chinese hamster ovary cells, but emerging expression platforms, including in bacteria, yeast, fungi, and tobacco plants, may help to reduce COGS [25]. The use of perfusion bioreactors and continuous processing, as opposed to batch processing, alongside product optimization, could also bring down costs [24, 25, 28]. Upstream investment in antibody engineering and cell line optimization, along with VLS manufacturing, could also drive down COGS further.

Investments in R&D to simplify administration (e.g., avoiding intravenous delivery by prioritizing subcutaneous formulations), lower dosing requirements, and decrease frequency of administration would reduce the costs faced by end users and health care systems. Evidence on the relative impact and scalability of different upstream and downstream interventions to reduce COGS is needed. Engaging with funders, developers, and manufacturers to seek their upfront commitment to supporting complementary technologies and proof-of-concept activities will help ensure process improvements are adopted in the development of future mAb products.

*Identifying and investing in trailblazer mAbs*. Pilot projects to expand access to mAbs could focus initially on a small number of ‘trailblazer products’, which could provide early impact and proof-of-concept for optimized product profiles and business model approaches to expand access. Candidates for a ‘trailblazer’ project could be NCD or ID mAbs that are already approved in HICs and align with PPCs but are not yet affordably and equitably available, such as nirsevimab for RSV, or those in clinical development with promising use cases, such as mAbs for malaria and HIV (Box 1).

**Box 1. Case studies of potential trailblazer ID mAbs**

mAbs for the prevention of malaria

Roughly 249 million cases of malaria occurred worldwide in 2022, resulting in an estimated 608,000 deaths, most of which were among children living in sub-Saharan Africa [29]. There are currently multiple prevention options for malaria recommended by WHO targeting children younger than 5 years of age in endemic areas, including malaria chemoprophylaxis using a combination regimen of sulfadoxine–pyrimethamine plus amodiaquine, and the R21/Matrix-M and RTS/S vaccines, both of which were recently recommended by WHO [30, 31]. Passive immunization with mAbs could offer an important complement to these options, providing immediate protection, less variable immune responses than those generated by vaccines across all age groups, and affording a longer-acting alternative to chemoprophylaxis to enhance compliance and reduce burden on the health care system.

Several mAbs for malaria prophylaxis are in preclinical, Phase 1, and Phase 2 development [32–34]. A Phase 2 trial evaluating the safety and efficacy of a one-time intravenous infusion of the CIS43LS mAb, developed by the US National Institute of Allergy and Infectious Diseases (NIAID), found that the antibody was safe and provided up to 88.2% protection from malaria infection among healthy, non-pregnant adults in Mali over a six month period with a dose of just 5mg/kg [32]. L9LS–a next generation more potent mAb, also developed by NIAID–demonstrated 80% protection against controlled human malaria infection in a Phase 1 trial when delivered by a single subcutaneous injection [33]. A single subcutaneous dose of 300mg of L9LS in 6–10 year olds in Mali provided 77% protection against clinical infection over 6 months [35], enabling relatively low COGS. Ongoing studies with L9LS in infants and children in Kenya will determine the efficacy of L9LS over 6–12 months against perennial transmission. Other malaria candidates are also in development targeting different stages of the parasite lifecycle.

mAbs for the prevention of RSV

RSV caused an estimated 101,400 deaths in children under 5 in 2019 [36]. Palivizumab, approved in 1998, was the first RSV mAb recommended for use in the initial 1–2 years of life in infants at particularly high risk of severe RSV [37]. Its intensive dosing schedule, which requires 4–5 monthly injections during the RSV season, coupled with high pricing and limited registration in the highest RSV incidence settings in LMICs limit its potential impact [1].

Optimized mAbs for RSV prophylaxis have now entered the market. Nirsevimab, approved by the EMA in 2022, is a single-dose, long-acting preventive option that can provide protection for the duration of RSV season. Although nirsevimab is now recommended for broad use in the EU and US [38, 39], due to its high price and limited availability it remains inaccessible in many areas, especially in LMICs [40]. Other long-acting RSV mAbs are in the pipeline, including RSM01, which is being developed by the Bill & Melinda Gates Medical Research Institute (GMRI) [41], and clesrovimab, being developed by Merck [42]. Moving forward, the use case and optimal implementation scenarios for RSV mAbs must be evaluated in the context of a shifting prevention landscape, which includes the recently approved maternal RSV vaccine for use during pregnancy to prevent lower respiratory tract disease (LRTD) and severe LRTD RSV in infants from birth through 6 months of age [43].

Broadly neutralizing antibodies for prevention of HIV

Broadly neutralizing antibodies (bnAbs) are antibodies that neutralize a wide range of different HIV-1 strains and are now being investigated for use in HIV prevention, treatment, and cure [44] for adult and infant indications [45]. In particular, bnAbs have attracted interest for their potential as a long-acting prevention option for infants at risk of being infected with HIV [46], a population for which there is a clear need for new prevention tools. Over 1.2 million newborns are exposed to HIV each year [47] and there were 130,000 new HIV infections in children in 2022 [47], 70% of which arose from scenarios in which the HIV prevention cascade wasn’t fully delivered [48]. Most pediatric transmission occurs during the perinatal and breastfeeding periods meaning that a short-term preventive intervention during this window could deliver life-long benefit.

Data from Phase 1 studies of multiple bnAbs suggest good safety and pharmacokinetics in infants, and infant macaque models suggest promising potential for use both in prevention and post-exposure prophylaxis [49–51]. Modeling suggests bnAb prophylaxis for 18 months in HIV-exposed infants could reduce vertical HIV transmission by up to 42% relative to the standard of care alone, depending on the setting, and would be cost effective or cost saving [52]. Quarterly or twice annual delivery of HIV bnAb combinations through subcutaneous or intra-muscular administration are now being pursued. If effective, HIV preventive bnAbs could mitigate adherence challenges with daily oral alternatives and support ease of supply chain and delivery through integration into the standard Expanded Program on Immunization schedule.

*Investing in innovative trial designs*. The massive research efforts set up in response to the coronavirus disease (COVID-19) pandemic have illustrated the potential of platform trials [53, 54], which evaluate multiple product candidates in parallel, creating efficiencies [55]. Platform trials could support ID mAbs development by allowing more effective use of clinical trial infrastructure and resources. However, risk sharing, regulatory guidance, robust statistical inputs, and strong alignment across stakeholders will be critical to build buy-in among product developers and regulators for platforms trials. Linking platform trial sponsors to local/regional mAb biosimilar manufacturers could allow an ‘end-to-end’ approach to support regional development and supply for mAb-based innovations.

### Intellectual property, licensing, and technology transfer

#### Challenge

The number of manufacturers, manufacturing capacity, and market competition are lower for biologics than for small-molecule medicines. Current global capacity for mAbs manufacture in mammalian cells is estimated at 30 tonnes per year, however it is projected that mAbs for the prevention of IDs in the pipeline could require tens of tonnes of additional annual manufacturing capacity. Even more could be required for the deployment of mAbs for pandemic response [25]. Limited competition in the mAbs market has contributed to high prices, supply insecurity, and limited access [56]. Global manufacturing capacity for mAbs needs to be significantly expanded and diversified to meet projected needs for ID mAbs and to accommodate the ever-increasing number of mAbs used for NCDs.

#### Key enablers

*Early planning for voluntary licensing and technology transfer*. Increasing the number of manufacturers for ID mAbs could expand global manufacturing capacity, decrease prices through competition, and increase supply security. Voluntary licensing, a mechanism wherein patent holders allow additional manufacturers to produce and sell generic or biosimilar versions of patented medicines in defined territories before patent expiry [57], could be a key enabler of expanded competition and could ensure supply in instances in which an innovator is not interested in pursuing commercialization of a product. Although still rare in the NCD and mAbs spaces, voluntary licensing has steadily become an expectation for innovations targeting infectious diseases disproportionately affecting LMICs, such as HIV and tuberculosis (TB) [56–59]. In designing voluntary licensing strategies, ensuring that licensing territories align with public health burden is key.

Technology transfer is a critical enabler and should include documentation and process know-how, as well as expert human resources, training, and the physical transfer of necessary materials to help reduce the timelines and costs for the development of biosimilar mAbs [60]. In particular, sharing of cell lines and transfer of manufacturing processes will significantly facilitate and accelerate voluntary licensing strategies [60].

*Supportive policy environments*. LMIC governments can support increased local mAbs manufacturing capacity by, among other things, implementing supportive industrial policies. Examples of such approaches can be seen in government policies in Brazil and Türkiye which support public-private partnerships for biologic manufacturing [61–64], and in strategies aimed at supporting pharmaceutical self-sufficiency, as advanced by the Ministry of Health of Indonesia [65], the Department of Biotechnology of India [66, 67], and the Federal Committee for Protection from Sanitary Risks in Mexico [68].

Expanding the manufacturing base for ID mAbs in LMICs can be expected to have a knock-on effect of supporting increased local and regional R&D capacity. However, in addition to this, LMIC researchers working on R&D relevant to ID mAbs can be supported through directed funding policies and research exchanges that engage LMIC-based researchers as equal collaborators.

The WHO influenza technology transfer initiative, which facilitated technology transfer to 13 developing country vaccine manufacturers over 2006–2019, identified a few central lessons learned: 1) the importance of whole-of-government and multisectoral coordination; 2) that collaboration goes beyond simply increasing manufacturing capacity; and 3) the importance of long-term planning, support, and vision [69]. Likewise, the Platform for Harmonized African Health Products Manufacturing (PHAHM; previously Partnership for African Vaccine Manufacturing) Framework for Action highlights regional R&D capacity as critical [70], alongside support for a sustainable regional mAbs manufacturing ecosystem [71]. These efforts complement other LMIC manufacturing capacity efforts, such as the mRNA Technology Transfer Programme [72] and the Regionalized Vaccine Manufacturing Collaborative [73].

*Incentive mechanisms and conditionalities*. While several companies have been willing to share patent rights to enable generic manufacture in LMICs, in some cases additional incentives may be needed to encourage the sharing of IP and technology transfer on the part of innovators. Incentives could be monetary [74], based in conditionalities (for example, as part of grants or funding contracts), or could involve political pressure. Governments and funders can support voluntary licensing and technology transfer for mAbs through legislation, policy, financing, and other incentive structures. One example of this is in Brazil, where government technology transfer policy has supported local manufacturing of several mAbs, including adalimumab, certolizumab, golimumab, palivizumab and tocilizumab [61, 75].

Compulsory licensing, a mechanism by which governments or courts can remove IP barriers, could be used to expand local production of mAbs in instances in which voluntary measures have failed and there are pressing health needs [56]. However, the potential impact of compulsory licenses is curtailed by the lack of technology transfer provisions and the requirement to seek compulsory licenses on a country-by-country basis.

*Supporting expanded regional manufacturing capacity*. Increasing mAbs manufacturing capacity in a broader range of LMICs could offer important benefits, such as strengthened regional supply security and enhanced political ownership. R&D and manufacturing capacity could be supported simultaneously by establishing local or regional mAbs development hubs. Such hubs have previously been created, including for palivizumab, influenza vaccines, and vaccine adjuvants [1, 12]. A ‘hub-and-spoke’ model (Box 2), like the one currently employed in the mRNA Technology Transfer Programme [76], could also be explored as a model for increasing mAbs manufacturing capacity in LMIC settings.

**Box 2. The ‘hub-and-spokes’ model for technology transfer**

A technology transfer hub brings together all the elements needed to establish a technology (including know-how, data, details on manufacturing processes, IP, and training) in a single place and then transfers the fully validated manufacturing process, or platform, to multiple users. Recipients (‘spokes’) of technology transfer from ‘hubs’ are equipped to manufacture specific products and to enable their long-term sustainable operation by training local staff, establishing supply chains for consumables, and supporting the development of independent funding streams, including through commercial sales. The entire network can also benefit from “spokes” reciprocally sharing any improvements that they make.

### Regulatory pathways and strategies to facilitate mAbs availability

#### Challenge

The majority of mAbs are not registered in lower-income countries: less than 10% of mAbs approved in the US and Europe are registered in Africa [1]. Nearly two decades after its initial approval, less than 10% of national essential medicines lists in LMICs include originator trastuzumab [77]. Complicated regulatory approval pathways and failure of product developers to apply for regulatory approval have delayed access to mAb-based innovations in LMICs [78–80].

The regulatory environment for biosimilars is more complex than that for small molecules. Unlike generic small molecules, which are exact copies of originator products, biosimilars are non-identical products that must demonstrate comparability with an already approved biologic medicine (the reference product) with no clinically meaningful difference in terms of efficacy and safety [81,82]. For biologics, manufacturing an identical product is not possible without access to the originator’s cell lines and details of their manufacturing processes, which are often proprietary [83].

#### Key enablers

*Supporting regulatory collaboration and mutual recognition*. Regulatory agencies with extensive experience in biologics, such as the US FDA and European Medicines Agency (EMA), together with the WHO Prequalification of Medical Products Programme, have established mechanisms that could be leveraged to support other regulators in reviewing the dossiers of ID mAbs for regulatory approval. WHO Prequalification and the EMA’s EU-Medicines for all procedures may provide important pathways for accelerating regulatory approval of ID mAbs for use in LMICs by facilitating joint reviews and the sharing of assessment reports [84, 85]. Strengthening regulatory networks with mutual recognition–through platforms such as the African Medicines

Agency/African Vaccines Regulatory Forum (AVAREF) and WHO’s Collaborative Registration Procedure (CRP)–could also facilitate faster and broader approval of new mAbs [86].

*Regulatory enablers*. Establishing biosimilarity based on pharmacokinetics, preclinical, and other data for an approved reference product could minimize the need to undertake efficacy trials that could otherwise pose a potential barrier for would-be biosimilar manufacturers. Greater use of *in vitro* pharmacodynamic markers, when available, and other non-clinical data for the ‘bridging’ of efficacy from a reference product could facilitate regulatory agencies’ approval of ID mAbs, reducing costs and timelines for biosimilar development [87, 88]. Where there is full technology transfer for a particular approved mAb product, the manufacturer should explore with regulatory agencies whether reduced testing requirements could be acceptable to support approval, relative to those required for an entirely new biosimilar.

Biosimilar developers frequently face challenges and high costs in obtaining an originator product for use as a reference product [60, 89]. Encouraging originator companies to provide products for use in comparative testing on reasonable terms (e.g., as part of a voluntary licensing agreement) would significantly facilitate biosimilar development and could be included as an access-oriented condition in R&D grants. Use of an approved biosimilar, where available, as the reference product instead of an originator product can also help reduce costs [90].

### Market shaping, de-risking, and demand creation

#### Challenge

To succeed, business models for the development and manufacture of ID mAbs will require mechanisms to recoup expenses. However, low, unpredictable, or fragmented demand, and a lack of high-income market for many IDs may limit commercial viability. High costs and a lack of clear financing and procurement pathways may limit uptake, which can hinder sustainability and could deter the entry of biosimilar companies into the ID mAbs market.

*Market shaping and de-risking incentives*. ‘Push’ funding, in the form of grants and subsidies, is key to enable R&D for ID mAbs for which the commercial market may be limited [91]. Additionally, ‘pull’ financing mechanisms, economic incentives that materialize once a milestone is achieved, have been widely recommended to spur antibiotic development [92, 93]. Similar instruments could be designed to incentivise the development and deployment of mAbs for IDs. Examples of ‘pull’ incentives include advanced market commitments, whereby a developer is guaranteed a certain procurement volume, or a market entry reward upon developing a product meeting certain criteria. Both ‘push’ and ‘pull’ funding can have a multiplier effect by stimulating private sector investments. They should, however, be linked with binding access provisions [94].

LMIC governments must play a leading role in facilitating adoption of ID mAbs into health systems. For example, where governments and national expert groups assess ID mAbs as appropriate for their health systems, adoption can be facilitated by inclusion in clinical guidelines, national procurement lists, national essential medicines lists, and/or national health insurance systems.

*Pooled procurement platforms*. Large procurement mechanisms have been established for drugs of significant global health importance, such as Global Fund’s pooled procurement platform, the Pan American Health Organization (PAHO) Strategic Fund, and UNICEF’s Supply Division. The African Centres for Disease Control and Prevention (Africa CDC) is also developing a new pooled procurement mechanism, linked to objectives of strengthening regional manufacturing [95]. Centralized procurement of mAbs through these and other pooled mechanisms, including EID stockpiles, can help consolidate demand, increase negotiating power, and facilitate broad geographic availability, particularly for small-volume mAbs. These approaches have already been used for trastuzumab and tocilizumab [96, 97]. Pooled procurement of prioritized mAbs could be combined with a ’portfolio’ manufacturing approach to provide a comprehensive supply/demand strategy for mAbs access (*see Table 2*).

*Demand forecasts and creation*. Detailed LMIC market intelligence–including understanding of regional priorities, validation of demand forecasts, and defining willingness-to-pay thresholds–will be necessary to attract R&D and manufacturing investments. Close cooperation between manufacturers, LMIC governments, and other funders will be important for accurate forecasting. Committed funding in the form of volume guarantees or procurement subsidies for products that are critically needed could help mitigate start-up risks.

Regional pooled procurement initiatives, such as those mentioned in the previous section, and surveillance networks, including the ASEAN Public Health Emergency Coordination System (APHECS) and the Eastern Mediterranean Public Health Network (EMPHNET), can strengthen demand forecasting and supply management systems [98–100]. Alongside these efforts, the philanthropic sector is also making important investments in ‘bottom-up’ mechanisms for characterising and strengthening demand for ID mAbs [26].

Awareness raising and advocacy will be important to garner political will and support integration of mAbs into existing financing, procurement, and delivery mechanisms. Community and civil society engagement to bolster mAbs health literacy will help support demand creation and uptake of new ID mAbs into health systems.

## Tailored business models for ID mAbs

To guide the design of new strategies and business models for ID mAbs, we identify three disease-market ‘archetypes’, each of which is associated with different combinations of market challenges and could benefit from tailored enabling strategies (*See Table 1*): emerging and re-emerging infectious diseases (EIDs), e.g., Ebola virus and “disease X” (unknown future pathogen); IDs with a disease burden predominating in LMICs, e.g., malaria, dengue, and rabies; and IDs with a significant burden in both HICs and LMICs, e.g., RSV and HIV:

1. *Emerging and re-emerging infectious diseases (EIDs)*: Public sector, philanthropic, or innovative financing approaches, for example ‘subscription’ mechanisms or publicly subsidized manufacturing, are likely needed to support the development and supply of EID products, particularly those that have limited pandemic potential or relevance for biodefense [62]. Stockpiles of investigational products that have advanced through Phase 1 development could expedite clinical development of EID products by enabling rapid deployment for efficacy evaluation as outbreaks occur [101]. Post-licensure stockpiles can likewise create supply security for EID mAbs with unpredictable demand.
2. *IDs with disease burden predominating in LMICs*: Product development partnerships (PDPs), non-profits that partner with public and private sector entities to drive product development for commercially neglected diseases, can be catalytic in de-risking R&D and spurring investment for products with profiles and business model approaches geared toward LMIC needs. To date, PDPs have been successful at developing and bringing to market new treatments for TB [102], neglected tropical diseases [103], HIV prevention [104, 105], and malaria [106–108]. PDPs could link with local research, development, and manufacturing initiatives to foster a sustainable ecosystem for biologics and biosimilars regionally. Market-shaping levers (see above) can incentivize private sector actors to invest in R&D for ID mAbs and to collaborate in a manner that supports broad LMIC access.
3. *IDs with significant burden in both HICs and LMICs*: For ‘dual market’ mAbs, for which a commercial market exists in HICs as well as LMICs, efforts are needed to ensure resulting mAbs can be made broadly available at affordable prices. Across disease archetypes, but particularly for dual market mAbs, establishing a biosimilar market through voluntary licensing and technology transfer can help diversify supply, spur competition, and support affordable pricing. *(*See *Intellectual property*, *licensing*, *and technology transfer)*

**Table 1:** Market challenges, enablers, and potential business model strategies for mAbs in different ID-market archetypes.

ID-market archetype	Potential indications	Market challenges	Potential business models for increasing R&D	Potential business models for increasing access	Cross-cutting enablers
**1. Emerging and re-emerging infectious diseases (EIDs)**	Ebola virus Disease X (unknown future pathogen of pandemic potential)	• Unpredictable needs limited commercial markets and low volumes, except in the case of pandemics during which outbreak demand could outweigh supply capacity. • Regionally focused diseases that are commercially unattractive to multinational or global entities • National/ regional outbreak responses generally driven by governments and humanitarian organizations Stockpiling key for rapid deployed for outbreak response, but access to stockpiled products is often not equitable.	• Build evidence base for multiple use-cases, including as medical countermeasures (MCM) as well as for routine pre-exposure prophylaxis, post-exposure prophylaxis, and treatment to broaden potential market and incentivize R&D Develop platform manufacturing approaches that can be applied to both commercial and non-commercial mAbs indications to support portfolio diversification and cross-subsidization (*See Portfolio based business model*). • Develop and stockpile ‘libraries’ of promising investigational mAbs that have been taken through Phase 1 and are available to rapidly advance through clinical development as outbreaks emerge.	• Creation of a public sector, end-to-end investment model to support mAbs product development and access. • Subsidies or ‘subscription’ mechanisms that fund development and manufacturing of EID agents. • Establishing manufacturing capacity to meet regular demand in LMICs and surge capacity to address outbreaks or biological security threats, including investment in modular, decentralized manufacturing strategies that can meet regional outbreak needs. • Regional, national, or global stockpiles of mAbs for rapid deployment during outbreaks.	• Coordination among funders and other stakeholders to ensure end-to-end financing for new ID mAbs and to identify synergies and partnership opportunities. • Identification of potential trailblazer mAbs, in alignment with priorities and preferences from affected communities and regions. • An inclusive and needs-based approach to research and development. • Early-stage investments in product and manufacturing process optimization to support LMIC-friendly target product profiles and affordability. • Supporting innovative trial designs to facilitate both early- and late-stage clinical development, including clinical trial platforms in LMICs • Prompt integration of mAbs into WHO and National Essential Medicines Lists to support reimbursement. • Factoring access considerations early in the R&D process and requiring binding access terms and conditions for financing and collaboration models. • End-to-end, stage-gated ‘push’ (grant) funding, and innovative ‘pull’ funding mechanisms, such as market entry rewards. • Product development partnership models (particularly for innovator products) • Voluntary licensing and technology transfer, where needed in support of competitive diversification. • Access incentives and policies (such as use of TRIPS flexibilities) to enable biosimilar manufacture for LMIC markets. • Streamlined and harmonized regulatory requirements and pathways. • Market-shaping mechanisms to stabilize demand and supply dynamics, such as advanced market commitments. • Procurement approaches that pool demand and decrease risk for manufacturers. • High-quality demand projections based on key stakeholder consensus, incorporating market-shaping signals. • Awareness raising, community engagement, mAbs health literacy, and advocacy to garner political will and support integration of mAbs into existing policy, financing, and procurement mechanisms.
**2. Infectious diseases with high burden predominating in LMICs**	Malaria Dengue fever Chikungunya	Large potential user population but concentrated in LMICs and limited commercial market in HICs. At scale, demand could outweigh supply capacity. Without optimization, COGS may dictate a pricing floor that is unaffordable in many LMICs or limit the use-case.	• Criteria-based economic incentives for developers to optimize product profiles and manufacturing processes to support low cost of goods and ease of delivery in resource-limited contexts. • Evidence generation on epidemiology and impact to support investment decision making.	• Grants and other funding conditional on inclusion of access provisions • Technology transfer to enable biosimilar manufacture for LMIC markets. • Clear financing mechanism, market-shaping, and access roadmap early in development. Regulatory strategies to facilitate entrance of biosimilar manufacturers.	
**3. Infectious diseases with “dual markets” (significant markets in both HICs and LMICs)**	RSV HIV Pathogens prone to antimicrobial resistance, e.g., pathogens causing neonatal sepsis COVID-19	• Significant commercial market in HICs, but unaffordable or unavailable in most LMICs • Limited registration of originator mAbs in LMICs • Risk of diversion or HIC pricing impact from differential pricing may pose a barrier to industry engagement • Limited or delayed voluntary licensing to additional suppliers • mAbs not included in national essential medicines lists, and not integrated into national insurance schemes or public sector financing in LMICs • mAbs profiles not optimized for use in LMICs	• Applying strategies from archetypes 1 and 2 as relevant, including criteria-based economic incentives and evidence to support investment decision making.	• Establishing an LMICs market through voluntary licensing and technology transfer, tiered pricing or ‘second brands’ • Applying strategies from archetypes 1 and 2, including voluntary licensing, facilitated regulatory pathways, and early financing and access planning.	

For all market archetypes, public and philanthropic investments must be tied to binding access provisions, including affordability clauses and assurance of timely provision of volumes to countries in need. A roadmap for ensuring equitable access should be developed from the earliest stages of R&D to help coordinate and guide developers and funders along the entire product development pathway.

A portfolio-based strategy that bundles mAbs with different market profiles (e.g. mAbs for EIDs, mAbs for IDs, and mAbs for NCDs or other IDs with stable market profiles) has been proposed as a potential approach to foster a commercially viable business model for niche or low-volume mAbs, by cross-subsidizing production costs and by leveraging volumes across a range of products to support economies of scale (*See Table 2*). Further analysis of the economic viability, product portfolio strategy, start-up investments, and operational considerations for implementing a portfolio approach are needed.

**Table 2:** Portfolio-based business model.

Strategy	Potential indications	Market challenges addressed	Business model and enablers
**Portfolio approach**	Combined strategy to improve access to a selection of mAbs across the three categories above, and possibly also for NCD mAbs.	• Maintenance of manufacturing sites is very expensive. • Production capacity for mAbs for diseases with small, regionally focused markets might not be sustainable.	• 3–6 mAbs selected, as defined by regional or global priorities, representing a mix of product types, including mAbs for IDs with high burden of disease/high expected volumes (archetypes 2/3), mAbs for EIDs with niche markets/low volumes and high uncertainty (archetype 1), and mAbs for NCDs. • Some mAbs in the portfolio included on the basis of having an established commercial market and stable demand to provide a reliable income stream and cross-subsidise manufacturing overhead for other products in the portfolio that have limited commercial markets. • Interventions, including technology transfer and pooled procurement, to facilitate portfolio diversification and consolidate demand. • Investment in building or reserving manufacturing capacity to support portfolio needs, tied to access conditionalities.

## Conclusions: The way forward

mAbs have significantly improved health outcomes across diseases, however there is enormous global inequity in mAbs access. Realizing the potential of mAbs for global health will require targeted interventions along the product development continuum.

Numerous systemic challenges underly the gaps in R&D and access. Efforts to address these challenges must be cross-cutting and end-to-end, including early-stage product optimization and manufacturing innovations; access-oriented IP strategies, voluntary licensing, and technology transfer; strengthened regulatory pathways; and catalytic market shaping, de-risking, and demand creation strategies.

Novel business models and public-private collaborations are needed to advance this ambitious agenda. PDPs have demonstrated success in brokering viable business model strategies to advance health products for a range of neglected diseases, and likewise could be harnessed for ID mAbs. Integration with regionalization and localization agendas will be critical and will require strong collaboration with LMIC governments, regional entities, and affected communities early-on. Significant investments will be needed to support this vision and coordination among relevant funders will be important to ensure ‘end-to-end’ integrated access planning and financing.

As was seen in the scale-up of antiretroviral therapy for HIV treatment, demonstrating success with trailblazer mAbs could provide proof-of-concept and help define a roadmap for ensuring the affordability and accessibility of mAbs for IDs and beyond. This will require creativity, commitment, and a fundamental conviction that changing the status quo is possible.
